# Large-scale gene expression profiling data of bone marrow stromal cells from osteoarthritic donors

**DOI:** 10.1016/j.dib.2016.06.007

**Published:** 2016-06-14

**Authors:** Maik Stiehler, Juliane Rauh, Cody Bünger, Angela Jacobi, Corina Vater, Theresa Schildberg, Cornelia Liebers, Klaus-Peter Günther, Henriette Bretschneider

**Affiliations:** a University Centre for Orthopaedics & Trauma Surgery and Centre for Translational Bone, Joint and Soft Tissue Research, University Hospital Carl Gustav Carus at Technische Universität Dresden, Germany; b Department of Orthopaedics, Aarhus University Hospital, Aarhus, Denmark

**Keywords:** Bone marrow stroll cells, Osteoarthritis, Microarray analysis, Gene Ontology

## Abstract

This data article contains data related to the research article entitled, “in vitro characterization of bone marrow stromal cells from osteoarthritic donors” [1]. Osteoarthritis (OA) represents the main indication for total joint arthroplasty and is one of the most frequent degenerative joint disorders. However, the exact etiology of OA remains unknown. Bone marrow stromal cells (BMSCs) can be easily isolated from bone marrow aspirates and provide an excellent source of progenitor cells. The data shows the identification of pivotal genes and pathways involved in osteoarthritis by comparing gene expression patterns of BMSCs from osteoarthritic versus healthy donors using an array-based approach.

**Specifications Table**

**Table:** 

Subject area	*Medicine*
More specific subject area	*Orthopaedics*
Type of data	*Image*
How data was acquired	*Affymetrix® GeneChip Human Genome U133 Plus 2.0 Arrays (Affymetrix®, Santa Clara, USA), Affymetrix, NetAffx™ Analysis Center*
Data format	*Analyzed*
Experimental factors	*Bone marrow aspirates were obtained from the pelvic compartment of advanced-stage idiopathic hip osteoarthritic and age-matched healthy donors. After BMSCs isolation and expansion until subconfluency total RNA of BMSCs at passage 1 was analyzed.*
Experimental features	*Pivotal genes and pathways involved in osteoarthritis were examined by microarray analysis.*
Data source location	*Dresden, Germany*
Data accessibility	*Data are within this article*

**Value of the data**

• Gene expression patterns of BMSCs from osteoarthritic versus healthy donors were compared to identify pivotal genes and pathways figure out the exact etiology of OA.
• Data may serve as starting point to develop new therapies to treat OA.
• The data indicate well-characterized (clinical, radiological, cellular and subcellular data) osteoarthritic and healthy control donor populations.
• OA is a frequent comorbidity potentially influencing bone regeneration, therefore OA-BMSCs are of interest in the field of tissue engineering.

## 1 Data

**Fig. 1:**
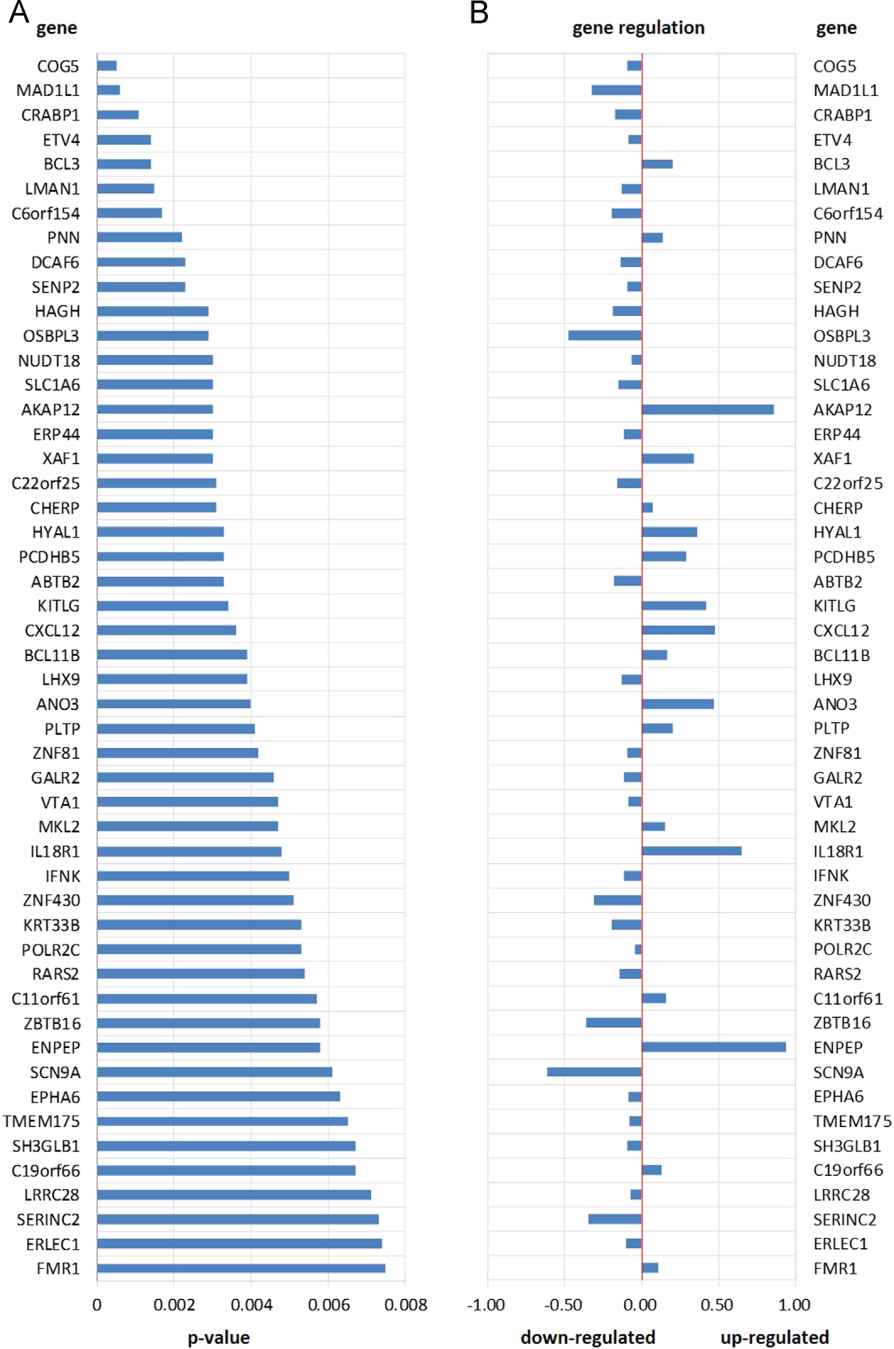
(A) Top 50 of the most significantly up and down regulated genes in BMSCs from osteoarthritic donors compared to healthy individuals arranged by *p*-value. (B) Difference in gene expression of the most significantly regulated genes; value>0 denote up-regulation, value<0 denote down-regulation.

**Fig. 2:**
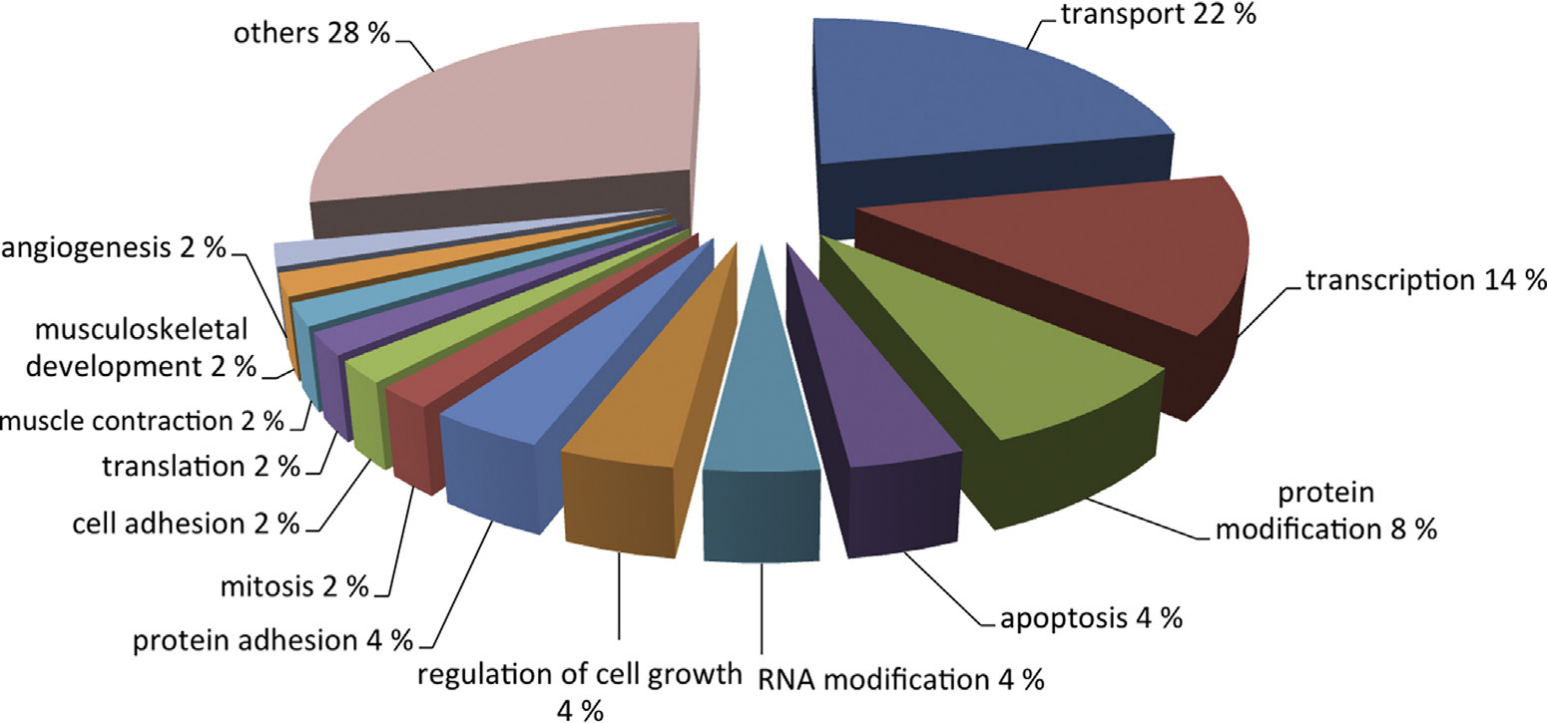
Percentaged Gene Ontology term enrichment of the 50 most significantly differentially expressed genes from osteoarthritic compared to healthy donors.

This data article provides a list of the most significantly up and down regulated genes in BMSCs from osteoarthritic donors compared to healthy individuals arranged by *p*-value (Fig. 1). The 50 most significantly differentially expressed genes between OA and control groups were analyzed with respect to Gene Ontology term enrichment (Fig. 1, Fig. 2.

## 2 Experimental design, materials and methods

Patients recruitment and characterization as well as cell isolation and cultivation is described in the research article entitled, “in vitro characterization of bone marrow stromal cells from osteoarthritic donors” by Stiehler et al., Stem Cell Research 2016 [1].

### 2.1 Microarray analysis

After cell expansion until subconfluency total RNA of MSCs at passage 1 was analyzed using Affymetrix® GeneChip Human Genome U133 Plus 2.0 Arrays (Affymetrix®, Santa Clara, USA) by AROS Applied Biotechnology A/S, Aarhus, Denmark. One MSC-pellet (passage 1, approx. 3–5·10^6^ MSC) per donor was shipped on dry ice to AROS core facility.

Raw data were processed by background correction, normalization, and robust multichip analysis followed by statistical analysis using “R” (The Comprehensive R Archive Network, https://cran.r-project.org 2010) and one-way ANOVA for gender-related or intergroup gene expression differences.

### 2.2 Gene Ontology analysis

A ranking with 690 intergroup differentially regulated genes was established using NetAffx™ Analysis Center (http://www.affymetrix.com 2010). Gene Ontology analysis was performed by DAVID (http://david.abcc.ncifcrf.gov 2011).
